# Carious status and socio-behavioral risk factors among 12 year-old children in South-Central region in Romania

**DOI:** 10.1186/s12903-023-03360-w

**Published:** 2023-09-06

**Authors:** Ruxandra Sfeatcu, Mariana Cărămidă, Ruxandra Sava-Rosianu, Marius Lupșa Matichescu, Atena Galuscan, Mihaela Adina Dumitrache

**Affiliations:** 1 Department of Oral Health and Community Dentistry, Faculty of Dentistry, “Carol Davila” University of Medicine and Pharmacy, 17–21 Calea Plevnei Street, Sector 1, Bucharest, 010221 Romania; 2 Translational and Experimental Clinical Research Centre in Oral Health, Department of Preventive Community Dentistry and Oral Health, University of Medicine and Pharmacy “Victor Babes”, Timisoara, 300040 Romania; 3 Research and Social Development Center, West University of Timisoara, Timisoara, 300223 Romania; 4 Social Research and Development Centre, Department of Sociology, Faculty of Sociology and Psychology, West University of Timisoara, Timisoara, 300223 Romania

**Keywords:** Children, Oral health, Caries prevalence, Health behavior, Dental caries, Risk factors

## Abstract

**Background:**

Regular screenings at children population level are necessary in order to assess communities’ need for education, prevention, as well as early detection and intervention programs The study aims to assess dental caries experience and oral health-related behaviors among Romania children from the South and Central region.

**Methods:**

The cross-sectional study conducted in 2019–2020 is part of the national survey “Romanian Oral Health Survey”, under the endorsement of the World Health Organization (WHO). The stratified, randomized and representative sample included 98 children of 12 years of age. The assessment included a clinical examination, using International Caries Detection and Assessment System (ICDAS) and the Oral Health Questionnaire for Children recommended by the World Health Organization.

**Results:**

Results revealed that the frequency of caries-free children among 12 year-olds was 36.7% and the mean DMFT was 2.89. Regarding the oral health-related habits, 38.8% of children brushed twice daily; the consumption of sugary foods multiple times per day has been frequently met and 11.2% at the age of 12 never visited the dental office.

**Conclusions:**

The findings revealed that caries prevalence was high and behaviors negatively influence oral health. The presented data are of utmost importance for Romanian policy makers to promote oral health education among children, to support consistent preventive oral health campaigns and to stimulate dental attendance, irrespective of children’ living areas or gender.

## Background

 Untreated dental caries remain the most prevalent health condition met at global level [1], with its peak prevalence observed in permanent dentition of 20–24 years old adults [2] and a second peak reached at the age of 70 [3]. Unfortunately, dental caries affect dentition from early stages in life, the peak prevalence of untreated caries among children with deciduous teeth being observed at the age of 5 years [2].

Despite its preventable characteristic and previous intensive efforts in oral health promotion under the World Health Organization (WHO) guidance [4], in the past 20 years dental caries prevalence decreased in high and upper-middle income countries opposite to lower-middle and low income countries where the prevalence increased [2]. A good practice model are countries like UK or Sweden where in 40 years of efficient and public health policies, the prevalence of dental caries decreased to a low level [3]. Thus, in the light of this background evolution and disparities, further actions are both needed and encouraged to control the risk factors, prevent and early detect [5]. Children are the priority target group when it comes to oral health promotion aiming primary prevention of dental caries [6, 7]. There are well-known risk factors that influence the development of dental caries. Behavioral risk factors such as improper oral hygiene routine, eating habits with frequently and rich in free sugars meals and snacks, improper fluoride exposure, as well as neglected and treatment/emergency-oriented visits to the dental office, have a negative impact on oral health in general and dental caries development, in particular [8, 9]. Therefore, prior to oral health promotion among children, the assessment of exposure to these risk factors through appropriate surveys are of utmost importance to detect the vulnerable population as well as the riskiest behaviors to be approached [7].

On the other hand, there is a clear correlation between dental caries and socioeconomic status, thus populations living in developing or underdeveloped countries have an increased risk for dental caries [2], as well as children from families with lower education level, underprivileged communities [10]. Untreated dental caries in children lead to a lower quality of life because of the dental pain and lost school days [11–13].

Regular screenings at children population level are encouraged in order to assess communities’ need for education, prevention, as well as early detection and intervention programs when it comes to caries risk. The WHO recommends standard examinations and surveys at a certain age among children: 6 years and 12 years old [14]. At the age of 6 years, permanent dentition starts its eruption process with first molars being vulnerable in the first year because of its lack of maturity in enamel mineralization [15]. Prevention through sealants and fluoridation is of utmost importance in high risk groups [16, 17]. At the age of 12 the process of exfoliation of primary dentition is close to its end and most of the permanent teeth are already erupted. Under these circumstances, children should get support through education and clinical intervention to minimize the risk exposure and ongoing progression of dental caries [3].

In Romania, the most recent assessment of dental caries experience at the national level has been developed between 2019 and 2020 through the “Romanian Oral Health Survey”; the results published in 2021 showed that the percentage of caries-free for 12 years old children is low, ranging between 26.26% in Bucharest, the capital city, and 7.50% in Banat area [18].

When it comes to oral health promotion and public health programs for dental caries reduction, there is a lack of consistency and continuity, at the time of this study no national oral health program was under implementation or progress.

The aim of the study was to assess the dental caries experience and its association with socio-behavioral risk factors among 12-year-old children from the South and Central region of Romania.

## Materials and methods

### Survey methodology

The present cross-sectional study was conducted in 2019–2020 as part of the national epidemiological survey “Romanian Oral Health Survey”, under the endorsement of the World Health Organization (WHO) that was sought to support the implementation of the research. The WHO Collaborating Center for Epidemiology and Community Dentistry in Milan, Italy offered guidance through the whole development of the survey. The research was implemented under the protocol approved by the Romanian Ministry of Health (Approval No. 3411/05.04.2018) and the methodology was presented to and accepted by the representatives of School Inspectorate from counties where the survey was applied, as well as each School Board from which the children had been selected to participate. Moreover, parents or legal tutors of children invited to take part in the research were informed in respect to the approved protocol, Declaration of Helsinki as well as the current European General Data Protection Regulation and those who agreed gave their written consent for children to enroll in the survey. Children were assessed according to the WHO Oral Health Survey Methodology, 5^th^ Edition [14], through clinical examination and questionnaire, described as it follows.

### Survey population

The sample in the present paper comprised of schoolchildren from the South-Central region, as a sub-group of the entire sample of the “Romanian Oral Health Survey “ research [18], applying WHO criteria for Oral Health Survey [14] to obtain a representative population.

At national level, the sample of 6 and 12-year old children was selected from registered schoolchildren population, based on a sample size calculation with 95% confidence level (± 3% sampling error), after stratification on both counties and administrative units; locality type (urban and rural), and randomization The selection of the participants was based on the total number of children enrolled in the schools. In Romania school is mandatory by law and it includes primary school, middle school and high school, from 0 to 12^th^ grade. Using schools as an environment where children in our target could be reached, the selection of the sample was based on the recruitment of a representative group students enrolled in the Romanian schools. Thus, from the total number of 4696 schools, there were selected 49 schools from all the 42 counties. In each selected school, respectively in each selected class, all the students were invited to participate. From the entire national-level sample of 1623 children included in the “Romanian Oral Health Survey “ [18, 19], 814 subjects formed the group of 6^th^ graders (who, in general, correspond to the age of 12 years) from all 42 Romanian counties [18]. The present paper focused in the South-Central region, thus it reports data from the 4 counties corresponding to this region and the sample included 98 subjects from 6 schools in the following counties: Giurgiu, Dâmbovița, Prahova and Brașov. In the sample selection process there were taken into consideration school level predictors that included, among others, sociological indices that were dependent on county-level variables related to life expectancy, education and income [20]. The counties selected in the present paper represent a region in Romania with a lower development level compared to other regions, in regards to social services, work productivity, workforce, incomes and investments. The Human Developmental Index for the region assessed in the present paper has a value of 0.783 that is lower than the average of our country. Romanian mean Human Development Index is 0.821, varying from 0.775 in North-East region and 0.932 in Bucharest-Ilfov (capital city of Romania and the areas near-by) (https://globaldatalab.org/shdi/table/shdi/ROU/?levels=1+4and;years=2021and;interpolation=0and;extrapolation=0).

The included participants were in 6^th^ grade, with a mean age of 12.21 years; 55.1% were girls and 53.1% living in urban areas.

### Clinical examination

The clinical assessment was performed according to the guidelines recommended by the WHO in the Oral Health Survey—Basic Methods, 5^th^ Edition [14]. The “Romanian Oral Health Survey” included the stage of calibration prior to the data collection, in order to apply the standardized protocol. Dentists that were part of research team were invited to participate in the calibration procedure, which was carried out by examining 21 subjects. The inter-examiner kappa ranged from 0.74 to 0.86 and the intra-examiner kappa ranged from 0.81 to 0.92. At national level, the 10 calibrated examiners were distributed in all regions, of which two were responsible for the South-Central region, described in the present paper.

Children selected to participate in the study were admitted to clinical assessment only if they presented the informed consent signed by the parent/legal tutor, along with the filled-in questionnaire distributed by the research team in advance. Oral examinations were conducted in schools, in classrooms, under the surveillance of a school representative, with children sitting and oriented to natural light, using headlights, oral examination kits and cotton rolls in order to remove the debris and dry the tooth surfaces. Clinical findings were recorded on specific charts, using the modified International Caries Detection and Assessment System (ICDAS) as well as International Caries Classification and Management System (ICCMS) Guide of Practitioners and Educators [21]. Due to the fact that this “Romanian Oral Health Survey “ research aimed to evaluate both the presence and the severity of the lesions in order to establish baseline data regarding the oral health status of the sample, there was chosen the ICDAS criteria that offers this advantage over the WHO caries diagnostic criteria. Therefore, all available teeth and surfaces on both primary and permanent dentitions were assessed and the two-digit code was used to mark the presence of both cavitated and non-cavitated lesions (second digit in the code, varying from 1 to 6), restorations and sealants (first digit in the code, varying from 1 to 8) or missing teeth (special codes). Because of the inability to air-dry the tooth surface, there was used the modified ICDAS criteria, thus caries codes 1 and 2 were recorded as “A” representing first visual changes in the enamel.

### Survey questionnaire

The socio-behavioral factors for oral health were assessed using the WHO Oral Health Questionnaire for Children, as recommended in the Oral Health Survey—Basic Methods, 5^th^ Edition [14]. English translators as well as education and psychology specialists implemented the Romanian version of the questionnaire after translation and revisions. The questionnaire included items related to socio-demographic parameters, parents’ level or education, oral hygiene behaviors, eating habits, previous dental office attendance patterns as well as fluoride exposure. The printed questionnaires were sent by the research team to schools to be distributed one week prior to clinical examinations to be filled-out by the children. The completed questionnaires were collected and attached to charts on the day of clinical oral assessment.

### Data analysis

The collected data was analyzed using Microsoft Excel Data Analysis program as well as HLM version 7.02, Scientific Software International for descriptive statistics of clinical findings and socio-behavioral parameters. When it comes to the correlation between variables, Spearman correlation coefficient was used, while the F-test was used for the comparison between categorical variables. Statistically significant level was considered as *p* < 0.05.

When it comes to the clinical findings, recorded ICDAS codes [21] were computed in order to report the DMFT indices [14]. Thus, DT (decayed-teeth) component was calculated using the recorded codes for cavitated carious lesions (second digit in ICDAS code equal or higher than 3), while the MT (missing-teeth) was calculated based on the special codes and FT (filled-teeth) counted restored teeth with the first digit in ICDAS codes higher than 2. According to previous research [22] regarding the correspondence between ICDAS and WHO caries assessment criteria, the ICDAS code 3 was considered the cut-off point for inclusion of the carious lesion in the D component of the DMFT/DMFS index.

## Results

### Oral health status

In the studied group, the frequency of caries-free children was 36.7% (Table 1). Children presented a mean number of 3.9 non-cavitated dental lesions and 3.14 cavitated carious lesions (Table 1). In regards to differences in caries distribution between genders, is has been observed that statistically significant more boys were caries-free at the age of 12 years, having also a lower mean number of cavitated carious lesions (DS) but higher number of incipient carious lesions compared to girls (Table 1). In rural areas it was observed a higher proportion of children with cavity-free dentition but an increased mean number of carious lesions among those affected by caries (Table 1). However, the differences between urban and rural areas did not reach the statistical significance (Table 1).

**Table 1:** Distribution of dental caries

	**Mean number of carious lesions (tooth surfaces)** **Mean (SD)**				**Caries-free children frequency** **N (%)**	
	**Non-cavitated carious lesions** **(ICDAS code 0A)**	*p*-value	**Cavitated carious lesions** **(ICDAS codes 3–6)**	*p*-value	**ICDAS code 0**	*p*-value
Female (54)	3.48 (3.66)	.237	3.69 (5.52)	.221	15 (27.2%)	0.042
Male (44)	4.43 (4.22)		2.48 (3.80)		21 (47.7%)	
Urban (52)	3.05 (3.23)	.025	3.13 (5.25)	.986	18 (28.8%)	.644
Rural (46)	4.86 (4.45)		3.15 (4.38)		18 (39.1%)	
Total (98)	3.90 (3.91)		3.14 (3.13)		36 (36.7%)	

The DMFT index had a mean value of 2.89 ± 2.29 among 6 graders (Table 2), with the DT component (untreated dental caries) prevailing. Thus, on average, 12 year-olds had 2.08 affected teeth on permanent dentition (Table 2). Restorative treatments were observed in a reduced number of teeth irrespective. No statistical differences were found when subjects were compared based on sex and residential area distribution. Although the differences were not statistically significant, it was observed a higher DMFT index and DT among children living in urban areas compared to rural areas (Table 2). Moreover, even though girls presented a higher DMFT, it was observed that it was due to the fact that they had a lower number of untreated dental caries (DT) as well as lower number of teeth extracted due to cavities (MT) and an increased number of restorations (FT) compared to boys (Table 2).

**Table 2:** DMFT indices and its components

	**Mean (SD)**							
	**DMFT**	**p-value**	**DT**	***p*** **-value**	**MT**	***p*** **-value**	**FT**	***p*** **-value**
Female (54)	2.96 (2.14)	.758	2.05 (2.46)	.873	.06 (.23)	.406	0.85 (1.59)	.403
Male (44)	2.81 (2.49)		2.13 (2.52)		.11 (.44)		0.56 (1.74)	
Urban (52)	3.01 (2.22)	.582	2.26 (2.41)	.454	0.12 (.37)	.301	0.63 (1.37)	.572
Rural (46)	2.76 (2.39)		1.89 (2.55)		.04 (.29)		0.82 (1.94)	
Total (98)	2.89 (2.29)		2.08 (2.48)		.08 (.34)		0.72 (1.65)	

### Oral health-related behaviors

Results regarding children oral hygiene habits showed that at least twice daily toothbrushing was performed by only 38.80% at the age of 12 years, while 32.7% brushed once a day and the rest of them seldom (Table 3). The frequency of toothbrushing was higher among girls and children living in urban areas (Table 3). When it comes to the products used for oral hygiene, the vast majority of children used toothpaste, the majority used the toothbrush, but dental floss is used only by 2% of children of 12 years of age (Table 4).

**Table 3:** Frequency of toothbrushing

Frequency	N (%)				
	**Total**	**Female**	**Male**	**Urban**	**Rural**
≥ 2 times/day	38 (38.8%)	26 (48.1%)	12 (27.3%)	21 (40.4%)	17 (37%)
1 time /day	32 (32.7%)	12 (22.2%)	20 (45.5%)	16 (30.8%)	16 (34.8%)
< 1 time/day	28 (28.5%)	16 (29.7%)	12 (27.2%)	15 (28.8%)	13 (28.2%)

**Table 4:** Oral hygiene products use

Type of product	N (%)				
	**Total**	**Female**	**Male**	**Urban**	**Rural**
Toothbrush	94 (95.90%)	53 (98.10%)	41 (93.20%)	49 (94.20%)	45 (97.80%)
Toothpaste	97 (99.00%)	54 (100.00%)	43 (97.70%)	52 (100.00%)	45 (97.80%)
Dental floss	2 (2.00%)	1 (1.90%)	1 (2.30%)	1 (1.90%)	1 (2.20%)

### Oral health-related eating behaviors

Fresh fruits and vegetables, which are non-sugar food products, thus with a reduced impact on cavity development, are consumed at least once a day by 51.8% of children (Table 5). On the other hand the consumption of products with a high sugar content, namely beverages, candies and cakes was declared by 1 in 4 children to be for a couple of times per day (Table 5). Among all sugar-containing food products assessed in the survey, the most frequently consumed (plenty of times per day) were those with the greatest negative impact on caries development, namely above-mentioned sugar-added food products that favors the cariogenity through their characteristics additional to the presence of sugar: starchy food (biscuits/cakes), acidogenic (beverages/soft drinks), low clearance (sweets/candies and sugary chewing gum) (Table 5). In addition, it was observed a frequent consumption, of at least 2 times per day, of starchy food, sugar-sweetened chewing gum and sweetened tea in higher proportions among girls and children living in urban areas while higher frequency of boys and participants from rural areas eat fruits and vegetables plenty of times per day (Table 5).

**Table 5:** Eating habits

**Type of food products**	***N*** ** (%)**				
	**Total**	**Female**	**Male**	**Urban**	**Rural**
Fruits/Vegetables					
** > 1 time /day**	41 (41.80%)	19 (35.2%)	22 (50.0%)	18 (34.6%)	23 (50.0%)
**1 time /day**	33 (33.70%)	24 (44.4%)	9 (20.5%)	21 (40.4%)	12 (26.1%)
** < 1 time /day**	24 (24.50%)	11 (20.4%)	13 (29.5%)	13 (25.0%)	11 (23.9%)
Biscuits/cakes					
** > 1 time /day**	26 (26.50%)	18 (33.3%)	8 (18.2%)	19 (36.5%)	7 (15.2%)
**1 time /day**	18 (18.40%)	11 (20.4%)	7 (15.9%)	10 (19.2%)	8 (17.4%)
** < 1 time /day**	54 (55.10%)	25 (46.3%)	29 (65.9%)	23 (44.2%)	31 (67.4%)
Soft drinks/fruit juice					
** > 1 time /day**	23 (23.50%)	12 (22.2%)	11 (25.0%)	11 (21.2%)	12 (26.1%)
**1 time /day**	20 (20.40%)	12 (22.2%)	8 (18.2%)	11 (21.2%)	9 (19.6%)
** < 1 time /day**	55 (56.10%)	30 (55.6%)	25 (56.8%)	30 (57.7%)	25 (54.3%)
Jam/honey					
** > 1 time /day**	2 (2.00%)	0 (0%)	2 (4.5%)	1 (1.9%)	1 (2.2%)
**1 time /day**	14 (14.30%)	8 (14.8%)	6 (13.6%)	5 (9.6%)	9 (19.6%)
** < 1 time /day**	82 (83.70%)	46 (85.2%)	36 (81.8%)	46 (88.5%)	36 (78.3%)
Chewing gum with sugar					
** > 1 time /day**	19 (19.40%)	12 (22.2%)	7 (15.9%)	13 (25.0%)	6 (13.0%)
**1 time /day**	10 (10.20%)	7 (13.0%)	3 (6.8%)	4 (7.7%)	6 (13.0%)
** < 1 time /day**	69 (70.40%)	35 (64.8%)	34 (77.3%)	35 (67.3%)	34 (73.9%)
Candy/sweets					
** > 1 time /day**	26 (26.50%)	17 (31.5%)	9 (20.5%)	14 (26.9%)	12 (26.1%)
**1 time /day**	21 (21.40%)	12 (22.2%)	9 (20.5%)	10 (19.2%)	11 (23.9%)
** < 1 time /day**	51 (52.10%)	25 (46.3%)	26 (59.1%)	28 (53.8%)	23 (50.0%)
Milk with sugar/honey					
** > 1 time /day**	4 (4.10%)	2 (3.7%)	2 (4.5%)	3 (5.8%)	1 (2.2%)
**1 time /day**	18 (18.40%)	13 (24.1%)	5 (11.4%)	9 (17.3%)	9 (19.6%)
** < 1 time /day**	76 (77.50%)	39 (72.2%)	37 (84.1%)	40 (76.9%)	36 (78.3%)
Tea with sugar/honey					
** > 1 time /day**	10 (10.20%)	7 (13.0%)	3 (6.8%)	8 (15.4%)	2 (4.3%)
**1 time /day**	17 (17.30%)	12 (22.2%)	5 (11.4%)	9 (17.3%)	8 (17.4%)
** < 1 time /day**	71 (72.50%)	35 (64.8%)	36 (81.8%)	35 (67.3%)	36 (78.3%)
Hot cocoa with sugar/honey					
** > 1 time /day**	5 (5.20%)	2 (3.7%)	3 (7.0%)	3 (5.9%)	2 (4.3%)
**1 time /day**	12 (12.40%)	7 (13.0%)	5 (11.6%)	6 (11.8%)	6 (13.0%)
** < 1 time /day**	81 (82.40%)	45 (83.3%)	35 (81.4%)	42 (82.4%)	38 (82.6%)

### Dental services utilization

Until the age of 12 years, 11.2% of 12 years old children (6^th^ Grade) never visited the dental office (Table 6). Among those who accessed dental services in the past 12 months, most frequently went only once (Table 6). Regarding the main reason for which the dental services are accessed in the previous 12 months, out of the children who previously went to the dental office, 43.1% went because of the presence of dental pain as the main symptom of a dental emergency. Assessed children were less oriented toward routine dental check-ups: 27.6% among 6^th^ graders (Table 6). There were observed higher proportions of children who either never went to the dental office or not at all in the last 12 months among boys and those living in urban areas (Table 6). In addition, among those who used the dental services in the previous years, late presentation, thus with a dental emergency, were as well seen in boys and urban children population (Table 6).

**Table 6:** Dental services utilization pattern

Behavior	N (%)				
	**Total**	**Female**	**Male**	**Urban**	**Rural**
**Frequency of dental visits in the last 12 months**					
None in the last year	18 (18.4%)	8 (14.8%)	10 (22.7%)	11 (21.2%)	7 (15.2%)
Once	23 (23.5%)	13 (24.1%)	10 (22.7%)	10 (19.2%)	13 (28.3%)
2 times	12 (12.2%)	6 (11.1%)	6 (13.6%)	8 (15.4%)	4 (8.7%)
> 2 times	23 (23.5%)	15 (27.8%)	8 (18.1%)	9 (17.3%)	14 (30.4%)
Never	11 (11.2%)	5 (9.3%)	6 (13.6%)	7 (13.5%)	4 (8.7%)
No answer	11 (11.2%)	7 (13%)	4 (9.1%)	7 (13.5%)	4 (8.7%)
Total	98 (100%)	54 (100%)	44 (100%)	52 (100%)	46 (100%)
**Main reason for dental visits in the previous 12 months**					
Routine check-up	16 (27.6%)	13 (38.2%)	3 (12.5%)	6 (22.2%)	10 (32.3%)
Treatment / treatment follow-up	8 (13.8%)	3 (8.8%)	5 (20.8%)	4 (14.8%)	4 (12.9%)
Pain (Dental Emergency)	25 (43.1%)	14 (41.2%)	11 (45.8%)	12 (44.4%)	13 (41.9%)
Don’t know/don’t remember	9 (15.5%)	4 (11.8%)	5 (20.8%)	5 (18.5%)	4 (12.9%)
Total	58 (100%)	34 (100%)	24 (100%)	27(100%)	31 (100%)

### Association between oral health-related behaviors and carious status

When it comes to the impact of oral habits on carious status, results showed a negative correlation between DT and the frequency of toothbrushing. Thus, the number of teeth affected by untreated cavitated lesions increased as the frequency of toothbrushing decreased, reaching the statistically significance in the group of 12 years old children (Table 7). There has not been observed a correlation between the D component and the reasons for dental visits (Table 8) as well as the frequency of consumption of food products high in sugar content (Table 9). However, the highest DT was observed among children who drink soft drinks and chew sugar-sweetened gum (Table 9).

**Table 7:** Mean DT related to the frequency of toothbrushing

**Frequency of toothbrushing**	More than once / day	Once / day	Less than once / day	**Spearman**’**s** **rho**	*p*-value
**12 year-old children**	1.26 (2.15) *N* = 38	2.40 (2.55) *N* = 32	2.85 (2.56) *N* = 28	-.247	.014

**Table 8:** Mean DT related to the main reason for dental visits

**Reasons of dental visits**	**Routine check-up**	**Treatment / treatment follow-up**	**Pain**	**Don**’**t know/don**’**t remember**	**No answer**	**F-test**	*p*-value
**12 year-old children**	1.75 (2.38) *N* = 16	0.50 (2.07) *N* = 8	2.00 (2.48) *N* = 25	2.44 (3.12) *N* = 9	-	0.965	.416

**Table 9:** Mean DT related to the frequency of sugary food products consumption

Frequency of sugar-added food products	> 1 time /day	1 time /day	< 1 time /day	rho	*p*-value
Biscuits/cakes	3.42 (5.67) *N* = 26	2.56 (4.77) *N* = 18	3.20 (4.50) *N* = 54	-.031	.759
Candy/sweets	2.96 (5.73) *N* = 26	3.52 (4.48) *N* = 21	3.08 (4.58) *N* = 51	-.020	.842
Soft drinks/fresh juice	4.09 (6.30) *N* = 23	3.35 (4.97) *N* = 20	2.67 (4.08) *N* = 55	.098	.338
Chewing gum with sugar	4.01 (6.54) *N* = 19	2.70 (4.52) *N* = 10	2.97 (4.38) *N* = 69	.029	.775

We conducted a multiple ordinal regression for each of our main outcomes (i.e., DT and DMFT), and we introduced predictors in stages (Table 10). Firstly, we introduced the demographic variables (i.e., gender and locality type), secondly, we introduced the frequency of toothbrushing, and in the last step we introduced the frequency of sugary food products consumption. This approach allowed us to compute the explained variance that can be attributed to each category of predictors, from one-step to another. Although the frequency of toothbrushing was the only significant predictor, we found that the explained variance almost doubled when from Step 2 to Step 3, when we introduced the frequency of sugary food products.

**Table 10:** Multiple regression analysis to assess the contribution of each type of predictor

Predictor	DMFT		DT	
	Estimate (SE)	Pseudo R-Square	Estimate (SE)	Pseudo R-Square
*Step 1*		Cox & Snell = .008 Nagelkerke = .008		Cox & Snell = .014 Nagelkerke = .015
Gender	.078 (.212)		.275 (.232)	
Rural/Urban	.154 (.212)		-.080 (.230)	
*Step 2*		Cox & Snell = .026 Nagelkerke = .026		Cox & Snell = .053 Nagelkerke = .054
Gender	.109 (.213)		.331 (.235)	
Rural/Urban	.159 (.212)		-.076 (.231)	
Frequency of toothbrushing	-.124 (.093)		-.202*(.099)	
*Step 3*		Cox & Snell = .065 Nagelkerke = .066		Cox & Snell = .093 Nagelkerke = .095
Gender	.162 (.219)		.392 (.243)	
Rural/Urban	.142 (.222)		-.064 (.243)	
Frequency of toothbrushing	-.151 (.096)		-.233*(,104)	
Biscuits/cakes	.001 (.120)		-.098 (.132)	
Candy/sweets	-.180 (.113)		-.105 (124)	
Soft drinks/fresh juice	.095 (.108)		.121 (.119)	
Chewing gum with sugar	.091 (.108)		.115 (.120)	

## Discussion

In the studied group, the majority of children had dental caries and were exposed to carious risk factors. With the results described in the present paper we aimed to assess the present oral health status and habits among 12 year-olds in the Romanian context of lack of consistent oral health promotion policies and to use it as a reference point for future initiatives and also for following-up.

Clinical assessment revealed the presence of dental caries, cavitated as well as non-cavitated lesions, at two thirds of children of 12 years of age. These values of the dental caries prevalence is close to the average values found at the national level reported by the researchers that conducted the national study of which the present results were part of [18, 19]. However, the high percentage of affected children are similar to the results found in a previous study conducted in Romania in 2011 [23] but are far from the WHO goals for the year 2000 and 2020 [24–26]. Moreover, the clinical examination of the oral status showed that children with a history of dental caries seldom benefit from restorative treatments, as the results showed low values of FT component and that the D3-6 component prevailed. Among 12 year-old children we found a mean value of the DMFT index of 2.89 that is double of the value set by the WHO goals for the year of 2020 of only 1.5 permanent teeth with a history of dental caries [26]. Compared to the results revealed by a national pathfinder study conducted recently in Hungary [27], a neighboring country with a socio-economic status similar to Romania, the DMFT values in our group were very close to those found in Hungarian children at the age of 12 years [27].

The high prevalence of untreated dental caries and the reduced number of restorations confirm the suboptimal utilization of dental service, taking into consideration that until the age of 12 years not all the children had at least one dental check-up. Globally, it is commonly accepted and recommended by the oral health professionals that the children should be offered a first dental visit after the eruption of the first temporary tooth, thus between 6 months and 1 year of age [9]. Moreover, American Academy of Pediatric Dentistry in 2004 introduced for the first time the concept of the dental home [28], which is defined as “relationship between the dentist and the patient, inclusive of all aspects of oral health care delivered in a comprehensive, continuously accessible, coordinated, and family-centered way” [28]. The dental home is recommended to be set as early as the first year of life and aims to monitor, guide and preserve children oral health [28]. Unfortunately, in the studied group, among children who visited the dental office in the past 12 months, almost half went because of the presence of dental pain, thus highlighting overdue dental visits.

When it comes to oral hygiene, only two thirds of children brush twice daily, as recommended and of utmost importance for prevention of dental caries [9]. The data analysis showed a negative correlation between the frequency of toothbrushing and the DMFT value: the less often children brushed, the higher the number of teeth affected by dental caries. The proportion of children enrolled in our study who brush twice daily is less than among Hungarian children [27] where half of the 12 year-olds respect the recommended frequency, and much less than among Sweden children, where 9 out of 10 children at the age of 5 years already have this habit [29]. Sweden is one of the countries where consistent public health measures applied in the oral health field decreased in 40 years the prevalence of dental caries among 4 years old children, from 83 to 38% [3].

Regarding the use of dental floss, a reduced number of children of 12 years of age used it to clean the interdental spaces. A recently published systematic review on the prevalence of dental floss use among children with deciduous dentition showed that 70% of children never floss until the age of 6 years [30] although it is recommended to initiate the use of dental floss as soon as two adjacent teeth get in contact during the eruption of the primary dentition [31].

Among the children assessment in the present study it was observed one quarter of children who consume sugary food and drinks multiple times during the day. For optimal oral health, it is widely accepted that the consumption of sugary products should be kept at a minimum, ideally not more than once a day. A recent systematic review that stands at the base of the updated WHO recommendations for reducing free sugars intake highlights a lower risk for dental caries when free sugar is consumed less than 10% of the total energy intake [32]. Specifically, for the case of children, a recently published paper on Brazilian children population, assessed on a prospective study, reported that from 6 to 18 years of age the increment of dental caries was 66% higher among children considered high sugar consumers compared to low consumers [33]. The positive correlation between the frequency of sugary products consumption and the DMFT index was also found at the national level among Romanian children enrolled in the pathfinder survey of which the present study was part of [18, 19].

The present study reports the latest data regarding oral health status among 12 year-old children in the South and Central region of Romania. The research was conducted according to the WHO oral health survey methodology [14] in regards to the sample selection (in order for the studied population to be representative), as well as to the assessment process (clinical examination was performed by calibrated dentists in order to respect the recommended standardized oral examination protocol in communities). However, the study has its limitations. The reported data regarding the carious status were based on clinical assessment only, with no additional radiologic investigation for a precise diagnosis. In addition, the oral examination was performed in the classrooms in schools, therefore without the appropriate illumination and air-drying otherwise offered by the dental chair for the proper screening of dental caries.

## Conclusion

In the studied group: oral clinical examination revealed that children’s dental health is highly affected, the prevalence of dental caries was high among 12 year olds and, on average, the number of teeth with untreated caries is high, exceeding the values set by the WHO goals for children oral health. In addition, habits that negatively affect children oral health were found with a high frequency among the participants in the study. Our findings reveal an increased need for both curative and preventive dental procedures The present epidemiologic data are of utmost importance for policy makers in the Romanian Ministries of Health and Education both to promote oral health education among children and parents and to support consistent preventive oral health campaigns, as well as to stimulate dental attendance from early ages, irrespective of children’ living areas or gender. Understanding the trends regarding dental caries prevalence and its risk factors distribution through recurrent surveys is essential for the adjustment of the strategies to community needs when it comes to oral health promotion. Initiatives such as oral health education classes in public schools curriculum, as well as campaigns conducted with the involvement of the health and education authorities or school dentists could use the data reported in the present study.

## Data Availability

The data presented in this study are available from the corresponding authors upon reasonable request.
